# Adipocyte-Specific Fatty Acid-Binding Protein (AFABP) and Chemerin in Association with Gestational Diabetes: A Case-Control Study

**DOI:** 10.1155/2021/5533802

**Published:** 2021-04-28

**Authors:** Maryam Mosavat, Mitra Mirsanjari, Bashir A. Lwaleed, Maherah Kamarudin, Siti Zawiah Omar

**Affiliations:** ^1^Department of Obstetrics & Gynecology, Faculty of Medicine, University of Malaya, Kuala Lumpur, Malaysia; ^2^Mazandaran University of Medical Sciences, Emam Khomeini Hospital, Fereidonkenar, Mazandaran, Iran; ^3^School of Health Sciences at the University of Southampton, UK

## Abstract

**Background:**

Adipocytokines participate in regulating the inflammatory response in glucose homeostasis and type 2 diabetes. However, among these peptides, the role of adipocyte-specific fatty-acid-binding protein (AFABP), chemerin, and secreted protein acidic and rich in cysteine (SPARC) in gestational diabetes (GDM) has not been fully investigated.

**Method:**

The maternal fasting level of adipocytokines of 53 subjects with GDM and 43 normal pregnant (NGDM) was measured using multiplex immunoassay at 24–28 weeks, before delivery, immediate postpartum, and 2–6 months postpuerperium.

**Results:**

Higher levels of AFABP were associated with a 3.7-fold higher risk of GDM. Low chemerin levels were associated with a 3.6-fold higher risk of GDM. Interleukin-10 (IL-10) was inversely associated with the risk of GDM. SPARC had no association with GDM. AFABP was directly correlated to interleukin-6 (*r* = 0.50), insulin resistance index (*r* = 0.26), and body mass index (*r* = 0.28) and inversely correlated to C-reactive protein (*r* = −0.27). Chemerin levels were directly and strongly correlated with IL-10 (*r* = 0.41) and interleukin-4 (*r* = 0.50) and inversely correlated to insulin resistance index (*r* = −0.23) in GDM but not NGDM. In the longitudinal assessment, there were no significant differences in AFABP and chemerin concentrations of both studied groups.

**Conclusion:**

AFABP and chemerin were associated with a higher risk of GDM. These adipocytokines were related to insulin resistance, body mass index, and inflammation in pregnant women diagnosed with GDM.

## 1. Background

Adipose tissue plays a significant role in the pathophysiology and development of type 2 diabetes (T2DM) and gestational diabetes (GDM) [1–4]. GDM is a condition of abnormal maternal glucose tolerance that occurs for the first time in pregnancy and can be prevented by using insulin sensitizers [5]. GDM is associated with an increased risk for subsequent abnormal glucose tolerance later in life [6]. Adipose tissue is a metabolically dynamic tissue secreting adipocytokines (or adipokines) that possess endocrine and paracrine properties, which are involved in energy homeostasis, immune response and systemic inflammation, reproduction function, and blood pressure regulation [7]. Adipocytokines chemerin, AFABP (adipocyte-specific fatty acid-binding protein), interleukin-4 (IL-4), and interleukin-6 (IL-6) are enlisted in the regulation of insulin resistance and inflammation [8]. An increase in the level of several cytokines and in particular IL-6 has been reported in coronavirus disease-19 (COVID-19) infection and increased the risk of adverse prenatal outcomes such as gestational diabetes [9, 10]. The role of chemerin is a novel adipocytokine that plays a role in adipogenesis, energy metabolism, and inflammation [11]. Studies have shown that expression of both chemerin and its receptor was upregulated in fat tissue of animal model with obesity and T2DM, where its circulation was considerably related to characteristics of metabolic syndrome and obesity (e.g., circulating triglycerides, blood pressure, body fat content, and insulin resistance) in normoglycemic individuals [12]. Chemerin elevation has been shown to lead to insulin resistance in *in vitro* studies of human myocytes and to glucose reduction in *in vivo* studies of animals in obese mice [13]. Furthermore, vitamin D deficiency has been associated with higher levels of circulating inflammatory marker chemerin and low insulin sensitivity [14] and increase the risk of GDM [15, 16]. The recent finding reported the protective effect of vitamin D3 supplementation on GDM via improving the antioxidant and inflammatory status and decreasing circulating chemerin level [17]. In serial measurements in early, mid, and late pregnancy, chemerin levels have been related to a noticeably increase in late pregnancy compared to early and midpregnancy stages [18]. However, there are conflicting results regarding chemerin circulation during GDM with its level being shown to be either elevated [19–22], unchanged [23–26], or reduced [27].

Secreted protein acidic and rich in cysteine (SPARC), a multifunctional matricellular peptide of 43 kDa, is related to the extracellular matrix and expressed largely in the basal lamina [28]. SPARC manages cell functions including cell adhesion, differentiation, and proliferation [29], where the protein has a broad range of biological effects [30]. SPARC is highly expressed in subcutaneous fat, and its production and excretion in adipose tissue are affected by fat mass, insulin, and glucose [31]. SPARC with profibrotic effects participate in metabolic dysregulation in obesity [31]. High SPARC expression in adipose tissue leads to insulin resistance [32]. Furthermore, higher levels of this peptide have been linked to T2DM, diabetic retinopathy, and nephropathy [32, 33]. In a recent cross-sectional study on normal pregnancy and GDM, SPARC levels were significantly correlated with inflammation and dyslipidemia [32]. This recent study showed that SPARC independently represented insulin resistance in late pregnancy, suggesting its possible role in the pathophysiology of GDM. AFABP belongs to the fatty acid-binding protein family which is highly expressed in adipose tissue [34]. AFABP is the largest cytosolic protein of mature adipocytes, accounting for roughly 6% of total cellular proteins [35]. This protein acts as a significant regulator of systemic insulin sensitivity and lipid and glucose metabolism [36–38], where AFABP concentrations are directly associated with indicators of metabolic syndrome and vascular disease [34]. High fasting AFABP serum levels have been shown to predict the risk of metabolic and vascular morbidity and mortality in T2DM [34, 39]. It has also been reported that AFABP secretion promotes insulin resistance in male mice, where AFABP mediates the degradation of peroxisome proliferator-activated receptors (PPAR*γ*) in adipose tissue and consequently reduces the expression of insulin-sensitizing adiponectin [40]. AFABP has been correlated with insulin resistance and inflammation in T2DM and associated obesity [41]. Upregulation of AFABP in GDM mothers has been described in previous cross-sectional studies which may be linked with obesity and insulin resistance in pregnancy [42, 43]. A study on pregnant women has stated that fetal tissues are the main source of cord arterial serum AFABP [42]. The same study also suggests that in fetuses of pregnancy with GDM, AFABP values correlate with adiposity indicators [42].

To the best of the authors' knowledge, no study has defined the predictive value of chemerin, SPARC, and AFABP concentrations in the development of GDM. Therefore, this study has been designed to assess the association between the serum concentration of these adipocytokines and the development of GDM and to evaluate the circulation of these peptides throughout pregnancy and after delivery.

## 2. Method

The protocol of this study has been approved by the Scientific Review Committee of University of Malaya Medical Centre (UMMC) (reference number 1052.8, MEC ID 201402-0725), and written informed consent was obtained from all participants. The protocol of this study was published elsewhere [3, 4, 44]. Briefly, as the authors have described recently [3, 4, 44], pregnant women aged between 18 and 45 years, gestational age between 24 and 28 weeks, a singleton pregnancy, and intending to participate in our longitudinal study and delivery at UMMC were initially eligible to participate in this cohort study. Those women with multifetal pregnancy, history of pregestational diabetes or previous GDM, drug, smoke, and/or alcohol abuse, hypertension, heart disease, renal or liver disease, uncontrolled endocrine disease, or any medical conditions that would affect lipid and glucose metabolism were deemed not eligible to enter the study. At the end of the longitudinal study, pregnancy outcomes were abstracted by extracting information from medical records. Subjects from whom it was not possible to collect a fasting blood sample or those who developed any pregnancy complications such as preeclampsia, eclampsia, pregnancy-induced hypertension (PIH), preterm labour (<36 weeks), or postterm labour (>41 weeks and 6 days) were also excluded from the study. The maternal fasting samples were collected on four occasions:

Examination 1 (E1): 24–28 weeks of pregnancy (at the time of GDM screening).

Examination 2 (E2): prior to caesarean/vaginal delivery.

Examination 3 (E3): early postpartum; within 24 hours after delivery.

Examination 4 (E4): postpuerperium; within 2–6 months after delivery.

Screening for the diagnosis of GDM was performed by the 2-hour 75 g oral glucose tolerance test; fasting plasma glucose level greater than or equal to 5.6 mmol/L or 2-hour plasma glucose level greater than or equal to 7.8 mmol/L [45]. Serum levels of SPARC, chemerin, AFABP, IL-4, IL-6, IL-10, and CRP were measured using Magnetic Multiplex Sandwich ELISA assay (LXSAHM, R&D, USA). Based on the manufacturer's report the intra-inter-assay coefficient of variation (CV%) of the assays were as follows: adiponectin (5.6-9.2), chemerin (6.7-13.7), AFABP (4.7-13.8) and SPARC (5.0-12.0), IL-4 (6.3-10.27), IL-6 (5.2-9.6), IL-10 (5.4-10.73), and CRP [9–13]. Homeostasis model assessment index (HOMA-IR) was computed using the following formula: fasting serum glucose (mmol/L)/22.5 × fasting serum insulin (mIU/L). Data regarding HOMA-IR has been reported previously [3].

### 2.1. Statistical Analysis

Descriptive statistics were applied for qualitative variables (mean ± standard error (SE)). The parametric Student's *t*-test or nonparametric Mann–Whitney *U* test was used to compare differences between two independent groups. Binary and multivariate logistic regression was performed to explore associations between the studied peptides and GDM risk (odds ratio (OR; 95% confidence interval (CI)). A paired sample *t*-test or two-related samples Wilcoxon test was performed to assess longitudinal changes between different time points (using Bonferroni correction). The Spearman/Pearson correlation coefficient described the correlation between peptides and metabolic markers. The *p* value of <0.05 was considered as statistically significant. All statistical analyses were performed using IBM SPSS 26.0.

## 3. Results

Ninety-six pregnant participants comprised of 53 who were diagnosed with GDM and 43 with normal pregnancy (NGDM) were recruited in this study. The demographic characteristics of the subjects are presented in Table 1. At the time of Examination 1, no significant differences were observed for participants' age (*p* > 0.24), pregestational BMI (*p* = 0.40), gestational BMI (*p* > 0.88), gestational weeks (*p* = 0.72), family history of diabetes (*p* = 0.07), and parity (*p* = 0.88) between both the GDM and NGDM groups. As expected, the GDM group presented a higher fasting blood glucose level (*p* = 0.003) and 2 hours postprandial glucose tolerance test (*p* = 0.006), as compared to NGDM subjects. Those diagnosed with GDM presented lower IL-10 (*p* < 0.001) and IL-4 (*p* = 0.04) compared to the normal group. No significant difference was observed in the level of IL-6 and CRP.

Using logistic regression, there was no association between SPARC, IL-4, IL-6, and CRP concentration and GDM risk (Table 2). IL-10 was inversely associated with the risk of GDM (0.18 (95% CI: 0.07-0.45)). Serum AFABP was directly associated with GDM risk. Participants with the higher levels of AFABP (>10.09 ng/mL) had a 3.7-fold higher risk of developing GDM compared with the lowest level. However, this relationship was attenuated but remained significant after adjustment for confounders including maternal age, gestational weeks, and BMI (aOR 1.1, 95% CI: 1.00-1.22). Serum chemerin (OR 0.85 (95% CI: 0.73-0.98)) was inversely associated with GDM. In the crude analysis, participants with chemerin (<8.03 ng/mL) presented a 3.6-fold higher risk of GDM compared to participants with the >10.2 ng/mL (95% CI: 1.3-10.4). After adjustment for maternal age, gestational age, and BMI, the lowest tertile of the chemerin value remained a strong predictor for the diagnosis of GDM (aOR 4.5; 95% CI: 1.4-14.0) (Table 2).

Using Spearman/Pearson correlation coefficient, AFABP was directly correlated to IL-6 (*r* = 0.50), HOMA-IR (*r* = 0.26), BMI (*r* = 0.28), and CRP (*r* = 0.27). Serum chemerin level was directly and strongly correlated with IL-10 (*r* = 0.41) and IL-4 (*r* = 0.50) and inversely correlated to HOMA-IR (*r* = −0.23) in GDM but not NGDM.

The intra- and intergroup comparisons of adipocytokines are shown in Table 3. Over the pregnancy and postpuerperium, no significant difference was observed in the SPARC level between both studied groups. Levels of AFABP concentration were high in GDM just in E1 (*p* = 0.04) compared to NGDM. AFABP was in the lowest level in the first examination; however, with advancement in gestational age, its levels increased (*p* < 0.0001) and reached a peak in late pregnancy in both GDM and NGDM. Immediately after delivery, the level of AFABP decreased (*p* < 0.002) and this reduction continued slightly in postpuerperium. Serum chemerin was significantly low in GDM compared to NGDM (E1: *p* = 0.02). There was no significant difference between chemerin concentration of GDM and NGDM groups in late pregnancy and after delivery. In the longitudinal assessment, chemerin levels of the normal pregnant group decreased with pregnancy development (*p* < 0.05). This reduction continued slightly and reached to its lowest level in postpuerperium (E4). In contrast, there were no significant changes in chemerin concentration of GDM with progress in gestational age. However, its concentrations reduced slightly (*p* > 0.05) in E3 and reached to its lowest level (*p* < 0.05) in E4.

## 4. Discussion

In this study, we showed that higher circulation of fasting serum AFABP concentrations in the second trimester, at the time of GDM screening, was associated with an increased risk for the development of GDM. When we categorized AFABP concentration to the four quartiles, we noticed that participants of upper quartile with the AFABP level higher than 10.09 ng/mL were at risk of GDM about 3.7-fold higher than pregnant women in the lowest quartile (AFABP < 4.90 ng/mL). This relationship is attenuated and reduced to about 1.1 but remained significant when we adjusted for confounders including maternal age, gestational week, and BMI. Similarly, a study on pregnant women in their first trimester of pregnancy reported an association of GDM risk with higher quartile AFABP concentration [42, 43, 46]. AFABP is highly expressed in adipocytes and contributes to insulin sensitivity and energy metabolism. We further observed that AFABP levels were positively associated with HOMA-IR and BMI. Similar to this finding, AFABP has recently been proposed as a marker of metabolic syndrome in nonpregnancy states [34, 47, 48]. In another study, higher AFABP concentration in GDM was also noted to be an independent risk factor for increased insulin resistance [49]. The role of AFABP in association with insulin resistance, inflammation, and obesity in T2DM has been noted previously [41, 50]. Similarly, in this study, correlations between AFABP level and IL-6 and CRP indicated a proinflammatory role of this adipokine in GDM.

In our longitudinal assessment, we observed that serum AFABP levels in both pregnant groups increased with gestational age and then decreased immediately after delivery. This study is the first to evaluate longitudinal changes in serum AFABP in women with and without GDM during and after their pregnancy period. However, we only found one other study that reported a significant increase in AFABP from the second to the third trimester of pregnancy [43]. Based on a report by Ortega-Senovilla et al. [51], AFABP concentration of cord blood was shown to be higher than maternal level, suggesting that fetal tissues are the main source of AFAPB in cord blood. Hence, increased levels of AFABP in correlation with advanced gestational age and its reduction immediately after delivery implies a significant role of fetal tissues in AFABP production.

In the present study, the second trimester of GDM pregnancy is linked to lower chemerin concentration than normal pregnant controls. Subsequently, we found that chemerin concentrations ≤ 8.0 ng/mL are associated with a 3.3-fold increment in GDM risk compared to levels ≥ 10.2 ng/mL. This result was relatively unchanged after adjusting for BMI and maternal and gestational age. Furthermore, we observed that the chemerin level of GDM was inversely and independently correlated to HOMA-IR. Pregnancy is a complex of metabolic changes in early pregnancy, followed by insulin resistance [52]. It has been determined that GDM develops in the presence of the inability of pancreatic *β*-cells to induce sufficient insulin secretion and counteract insulin insensitivity of tissues during pregnancy [21]. An animal study has shown that chemerin and its receptors are substantially expressed in *β*-cells which are necessary for insulin secretion *in vitro* and *in vivo* [53]. Subsequently, chemerin deficiency causes glucose intolerance mainly due to increased hepatic glucose production and impaired insulin secretion [53]. Hence, a reduction in chemerin levels is associated with the development of GDM through decreased insulin sensitivity and attenuated anti-inflammatory capacity [21]. Similar to our study, Hare et al. [27] proposed that low chemerin levels in GDM may lead to insulin resistance and a higher level in normal pregnancy, which may provide a protective effect to decrease pregnancy-induced insulin resistance. Serum levels of chemerin may also be influenced by multiple factors in relation to inflammation and/or metabolic states related to obesity. Lower chemerin level observed in GDM subjects in the current study was formed in correlation with a reduction in IL-10 level. IL-10, an anti-inflammatory cytokine and an immunosuppressive, has been discussed as a key regulator for inflammatory cytokines. Moreli et al. [54], in a study on pregnant women, showed that glycemic mean ≥ 100 mg/dL is associated with a reduction in maternal IL-10 concentrations. Vitamin D level has been associated with circulating inflammatory markers and chemerin and low insulin sensitivity [14] and increase the risk of GDM [15, 16]. The protective effect of vitamin D3 supplementation on GDM through improving the antioxidant and inflammatory status and decreasing circulating chemerin level has been reported recently. In this study, as a limitation, we did not measure the level of vitamin. More studies are warranted to elucidate the relationship between vitamin D level and the inflammatory cytokines specially chemerin in GDM.

Interestingly, we also observed that pregnancy was associated with peak chemerin levels at 24-28 weeks of gestation. When pregnancy progressed, the differences between chemerin levels in both pregnancy groups diminished as serum chemerin levels in women with normal pregnancy decreased significantly, and the corresponding levels in GDM subjects remained relatively unchanged. We further noted an inverse independent correlation between chemerin levels and insulin resistance irrespective of maternal BMI levels. Our finding is in line with previous human and animal studies which revealed that chemerin levels are significantly reduced in circulating serum throughout pregnancy. This reduction has been shown to be inversely associated with a general increase in insulin resistance during gestation [27, 55]. Therefore, unchanged levels of chemerin in GDM subjects in the present study may be due to appropriate managing of disease with diet and/or insulin therapy. This may also explain the comparable serum chemerin levels and normal glucose tolerance in late pregnancy observed in subjects with GDM. In addition, during pregnancy, adipocytokines are produced not only by adipose tissue but also by the placenta [56]. An animal study by Garces et al. [55] has shown that the expression of chemerin mRNA was at its highest level at day 16. This subsequently decreased significantly towards the end of the pregnancy, hence describing an anti-inflammatory environment. Their study also indicated that reductions in levels of chemerin in both the placenta and maternal circulation may be necessary for adequate maternal-fetal immune interaction, essential for the normal progress of pregnancy. Within 24 hours after delivery, chemerin levels insignificantly reduced and remained statistically unchanged in both studied groups. However, there was a significant reduction in chemerin levels postpuerperium in the presence of normal glucose tolerance reestablishment, which challenge the probable role of chemerin in the progression of T2DM after pregnancy.

In summary, the findings of this study suggest that AFABP and chemerin are associated with increased GDM risk, with AFABP and chemerin levels being related to insulin resistance, BMI, and inflammation in women diagnosed with GDM. However, in the nonpregnant state, these peptides had no further contribution to the development of metabolic syndrome when glucose tolerance was achieved. Additional studies with a large sample size are desired to confirm the findings of this study.

## Figures and Tables

**Table 1:** Demographic characteristics of subjects, mean (SE).

	GDM	NGDM
Participants' age (year)	33.2 (0.6)	32.1 (0.8)
Gestational week	25.8 (0.2)	25.9 (0.2)
Pregestation BMI (kg/m^2^)	27.0 (0.8)	25.2 (0.7)
FBG (mmol/L)	5.0 (0.2)	4.2 (0.1)^∗^
2 hrs OGTT (mmol/L)	10.8 (1.5)	5.9 (0.1)^∗^
IL-10 (pg/mL)	1.2 (0.10)	4.0 (0.32)^∗^
IL-4 (pg/mL)	6.5 (0.31)	8.8 (1.67)^∗^
IL-6 (pg/mL)	2.8 (0.41)	2.6 (0.34)
CRP (ng/mL)	2.8 (0.86)	2.8 (0.33)

^∗^ *p* value < 0.05 significant difference between the two pregnancy groups.

**Table 2:** Association between adipocytokines and GDM risk (24-28 weeks).

		Unadjusted OR (95% CI)	Adjusted OR (95% CI)
IL-10 (pg/mL)		0.18 (0.07-0.45)^∗^	0.17 (0.06-0.48)^∗^
°*p* value		<0.001	0.001
IL-4 (pg/mL)		0.98 (0.96-1.01)	0.98 (0.95-1.00)
IL-6 (pg/mL)		1.03 (0.82-1.30)	1.03 (0.79-1.33)
CRP (ng/mL)		1.0 (1.0-1.01)	1.0 (1.0-1.01)
SPARC (*μ*g/mL)		0.44 (0.16-1.20)	0.12 (0.16-1.24)
AFABP (ng/mL)			
Quartile 1	*X* < 4.90	Referent	Referent
Quartile 2	4.90 < *X* < 7.57	2.02 (0.62-6.55)	1.92 (0.55-6.67)
Quartile 3	7.57 < *X* < 10.09	0.93 (0.29-3.0)	0.92 (0.27-3.11)
Quartile 4	*X* > 10.09	3.68 (1.06-12.77)^∗^	1.11 (1.00-1.22)^∗^
*p* value		0.04	0.03
Chemerin (ng/mL)			
Tertile 1	<8.03	3.6 (1.3-10.4)	4.5 (1.4-14.0)
Tertile 2	8.0 ≤ *X* < 10.2	1.2 (0.4-3.2)	1.1 (0.4-3.0)
Tertile 3	≥10.2	Referent	Referent
*p* value		<0.001^∗^	0.001^∗^

OR (95% CI) adjusted for maternal age, gestational age, and BMI. ^∗^*p* value < 0.05.

**Table 3:** Between- and within-group comparisons of adipocytokine.

	Examination (E1)	Examination (E2)	Examination (E3)	Examination (E4)
SPARC (*μ*g/mL)				
GDM	1.23 (0.07)	1.09 (0.06)^a^	1.08 (0.06)	1.03 (0.07)^a^
NGDM	1.37 (0.05)	1.11(0.05)^a^	1.06 (0.05)	1.00 (0.05)^a^
*p* value	0.11	0.80	0.80	0.68
AFABP (ng/mL)				
GDM	9.11 ± 0.69	13.57 ± 1.13^a^	12.47 ± 1.19^a,b^	10.87 (1.00)^b^
NGDM	7.25 ± 0.52	12.28 ± 0.91^a^	10.39 ± 0.79^a,b^	8.94 ± (0.68)^a,b^
*p* value	0.04	0.39	0.15	0.12
Chemerin (ng/mL)				
GDM	8.70 (0.46)	8.17 (0.38)	7.76 (0.32)	6.18 (0.35)^a,c^
NGDM	10.12 (0.35)	8.14 (0.34)^a^	7.74 (0.33)^a^	5.79 (0.25)^a,b,c^
*p* value	0.02	0.94	0.97	0.40

^a^ *p* value < 0.05 compared to Examination 1. ^b^*p* value < 0.05 compared to Examination 2. ^c^*p* value < 0.05 compared to Examination 3. ^∗^*p* value < 0.05 compared to NGDM.

## Data Availability

Data are available from the authors upon reasonable request.
